# A single amino acid substitution in the V protein of *Nipah virus* alters its ability to block interferon signalling in cells from different species

**DOI:** 10.1099/vir.0.82261-0

**Published:** 2006-12

**Authors:** Kathrin Hagmaier, Nicola Stock, Steve Goodbourn, Lin-Fa Wang, Richard Randall

**Affiliations:** 1 Centre for Biomolecular Sciences, University of St Andrews, The North Haugh, St Andrews KY16 9ST, UK; 2 Division of Basic Medical Sciences, St George's, University of London, London SW17 0RE, UK; 3 CSIRO Livestock Industries, Australian Animal Health Laboratory, Geelong, VIC 3220, Australia

## Abstract

The V protein of the paramyxovirus *Nipah virus* (NiV) has been shown to antagonize the interferon (IFN) response in human cells via sequestration of STAT1 and STAT2. This study describes a mutant of the NiV V protein, referred to as V(AAHL), that is unable to antagonize IFN signalling and demonstrates that a single amino acid substitution is responsible for its inactivity. The molecular basis for this was identified as a failure to interact with STAT1 and STAT2. It was also shown that NiV V, but not V(AAHL), was functional as an IFN antagonist in human, monkey, rabbit, dog, horse, pig and bat cells, which suggests that the ability of NiV to block IFN signalling is not a major constraint that prevents this virus from crossing species barriers.

In the last decade, zoonotic outbreaks of respiratory disease and encephalitis affecting humans, horses and pigs in Australia, Malaysia and Singapore have led to the isolation of two novel paramyxoviruses, *Hendra virus* (HeV) and *Nipah virus* (NiV) (Chua *et al.*, 2000; Murray *et al.*, 1995; O'Sullivan *et al.*, 1997). However, due to characteristic differences from other paramyxoviruses they have been assigned to a new genus, *Henipavirus* (Wang *et al.*, 2000). The natural hosts of both HeV and NiV are fruit bats (suborder Megachiroptera) of the genus *Pteropus* (Chua *et al.*, 2002; Halpin *et al.*, 2000). Neutralizing antibodies to NiV have also been found in an insectivorous bat (suborder Microchiroptera) (Yob *et al.*, 2001). In the initial HeV and NiV outbreaks, the viruses were transmitted from bats to humans by way of intermediate animal hosts, horses and pigs, respectively (Chua *et al.*, 2000). More recent outbreaks of NiV in Bangladesh and India have led to further human deaths and may have been a result of both bat-to-human and human-to-human transmissions (Butler, 2004; Chadha *et al.*, 2006; Enserink, 2004; Hsu *et al.*, 2004).

Similar to other paramyxoviruses, both henipaviruses have the potential to express multiple proteins, P, V, W and C, from the P gene by RNA editing and alternative translational initiation (reviewed by Lamb & Kolakofsky, 2001). The V and/or C proteins of various paramyxoviruses have been demonstrated to antagonize the interferon (IFN) system, part of the innate cellular immune response to viral infection, in several distinct ways (for recent reviews, see Horvath, 2004; Nagai & Kato, 2004; Stock *et al.*, 2005). NiV and HeV antagonize both IFN-*α*/*β* and IFN-*γ* signalling via the binding and sequestration of STAT1 and STAT2 in high-molecular-mass complexes (Rodriguez *et al.*, 2002, 2003). In other paramyxoviruses, the highly conserved cysteine-rich C terminus of the V protein is required to antagonize IFN signalling, but although henipavirus V proteins share this conserved C-terminal domain, it is dispensable for the sequestration of STAT1 and STAT2 by these viruses (Rodriguez *et al.*, 2004; Shaw *et al.*, 2004). The regions of NiV V that interact with STAT1 and STAT2 have been mapped to the N terminus of the protein, from residues 100 to 160 for STAT1 binding and a larger region comprising residues 100–300 for STAT2 binding (Rodriguez & Horvath, 2004; Rodriguez *et al.*, 2004). A similar study by Shaw *et al*. (2004) identified an overlapping area, residues 50–150, as sufficient for binding of STAT1 (Shaw *et al.*, 2004). These regions are also present in the NiV P and W proteins, both of which have been demonstrated to block IFN-*α*/*β* signalling and to bind STAT1 (Rodriguez *et al.*, 2002; Shaw *et al.*, 2004). The P, V and W proteins of NiV, as well as the C protein, which has a sequence distinct from other P gene products as a result of alternative translational initiation, also antagonize the IFN response in chicken cells (Park *et al.*, 2003).

As part of our comparative studies on how viruses circumvent the IFN response in different species, we were looking at the V protein of NiV. To this end, a cDNA encoding the NiV P gene was isolated from a plaque-purified NiV stock held at the Australian Animal Health Laboratory in Geelong, Australia (named NiV-AAHL for this study), which was derived from a human isolate. As the V mRNA is produced by insertion of a non-templated G residue into the P gene transcript at a conserved editing site, we introduced an extra G residue into the cDNA by overlapping PCR and cloned the open reading frame into the mammalian expression vector pEF.plink2 (Didcock *et al.*, 1999). A c-myc epitope tag was fused to the N terminus of NiV V to facilitate detection of the protein. Very surprisingly, given previously published work, IFN signalling assays in Vero cells showed that NiV-AAHL V [referred to as V(AAHL) hereafter] was unable to antagonize either IFN-*α*/*β* (Fig. 1a[Fig f1]) or IFN-*γ* signalling (data not shown).

Sequence analysis of the V(AAHL) gene and comparison with the V gene of the reference strain originally published by the CDC group (GenBank accession no. NP_112023; Chua *et al.*, 2000) and termed V(CDC) in this study revealed four nucleotide differences, two of which resulted in changes in the amino acid sequence of the V protein at residues 125 and 280 (Fig. 1b[Fig f1], open boxes). The P sequence from which V(AAHL) had been derived differed from the CDC sequence in the same four nucleotides. As V(CDC) was shown by Rodriguez *et al*. (2002) to block IFN signalling (Rodriguez *et al.*, 2002), we mutated residues 125 and 280 in V(AAHL) to the equivalent residues in V(CDC). The resulting protein, termed V*(CDC), was able to antagonize both IFN-*α*/*β* and IFN-*γ* signalling in Vero cells (Fig. 1a[Fig f1] and data not shown). Subsequently, these two amino acid changes were introduced into V(AAHL) individually, creating V(AAHL)-E125G and V(AAHL)-D280N. As shown in Fig. 1(a), only the mutant containing the E125G mutation was able to block IFN-*α*/*β* signalling. V(AAHL)-E125G was also able to antagonize IFN-*γ* signalling (data not shown). These results indicated that a single amino acid change from glutamic acid (E) to glycine (G) at residue 125 enabled V(AAHL) to block IFN signalling, suggesting that this residue plays a critical role in IFN antagonism by NiV V. Subsequent immunofluorescence experiments showed that constructs containing the E125G mutation, V(AAHL)-E125G and V*(CDC), prevented the nuclear translocation of STAT1 and STAT2 in response to IFN-*α* (Fig. 2a[Fig f2]) and also the nuclear translocation of STAT1 in response to IFN-*γ* (not shown), as previously demonstrated for V(CDC) (Rodriguez *et al.*, 2002), whilst V(AAHL) did not affect the distribution of STATs. Furthermore, residue 125 lies within the proposed STAT1- and STAT2-binding regions of NiV V (Rodriguez *et al.*, 2004; Shaw *et al.*, 2004), suggesting that the inactivity of V(AAHL) results from defective STAT binding. Indeed, co-immune precipitation experiments showed that the ability of V(AAHL) to bind STAT1 or STAT2 was dramatically reduced, whereas introduction of the glycine at residue 125 in V*(CDC) restored the binding capacity (Fig. 2b[Fig f2]). The G374A mutation also causes an amino acid change in the sequence of the C protein. However, although the C protein has been reported to be a weak IFN antagonist, its mode of action is unknown (Park *et al.*, 2003; Shaw *et al.*, 2004) and thus speculation about the possible influence of such a mutation on the function of C cannot be made at present.

NiV is able to replicate not only in bats, humans and pigs, but also in a number of other species such as hamsters, cats, dogs and horses (Hooper *et al.*, 2001; Middleton *et al.*, 2002; Wong *et al.*, 2003). We analysed the ability of NiV V to antagonize IFN signalling in cells from various species including Tb1 Lu lung epithelial cells from *Tadarida brasiliensis* (ECACC 90020805), which were of particular interest as the bat population of Southeast Asia is thought to be the reservoir of NiV. [It should be noted that *T. brasiliensis* is not among the bat species identified as the natural host of NiV. However, in addition to four species of fruit bat, antibodies against NiV have also been found in two insectivorous bats, which are more closely related to *T. brasiliensis* (Field *et al.*, 2001; Wacharapluesadee *et al.*, 2005; Yob *et al.*, 2001).]

The results from a panel of assays done in cell lines from different species are summarized in Fig. 3(a). V*(CDC) was able to inhibit IFN-*α* signalling in all species tested, i.e. cells from human, monkey, pig, dog, rabbit, horse and bat. The mutant V(AAHL) was inactive in all species tested except the bat cells, in which it retained some residual activity. A detailed representation of the results obtained in bat Tb1 Lu cells is given in Fig. 3(b). Due to the lack of appropriate tools, such as the sequences of the STAT genes from bat or effective antibodies against bat STAT proteins, we cannot at present provide an experimental explanation for this phenomenon. It might, however, point to a difference in the sequence of bat STAT proteins compared with the other species investigated here.

Single amino acid substitutions affecting IFN sensitivity have been observed in other paramyxoviruses and could reflect the ability of these viruses to cross species barriers (Chatziandreou *et al.*, 2002; Garcin *et al.*, 2002; Young *et al.*, 2001). It was therefore of considerable importance to know whether the G125E mutant was a natural variant that might play a role in the species-specific inhibition of IFN signalling or whether it had been generated during cloning of the V(AAHL) gene. We managed to obtain an earlier passage of the original human isolate of NiV(AAHL), as well as the plaque-purified stock that had been used to generate the V(AAHL) expression plasmid used in this study. We amplified the relevant region of the V genes that contained the position in question (positions 8–569, mRNA sense) by RT-PCR. The sequences obtained from both products had a guanine at position 374, which corresponds to a glycine at position 125 of the amino acid chain, and were thus identical to the sequence published for V(CDC). The most likely explanation for this discrepancy therefore appears to be that V(AAHL) represents a PCR artefact that arose during cloning of the expression plasmid. However, given the observation that the G125E mutation did not completely disrupt the function of the V protein in bat cells, we cannot rule out the possibility that, rather than being coincidental, the mutation might have been present in a small population of virus particles in the original stock. Whatever its origin, the results presented here demonstrate that a single amino acid in the STAT1/2-binding region of NiV V is critical for its ability to bind and prevent the relocalization of STATs in response to IFN stimulation. These findings underscore the importance of the N-terminal region of V for interaction with STATs (Rodriguez *et al.*, 2004; Shaw *et al.*, 2004).

Recently, a number of sequences from NiV isolated from humans and pigs, as well as a single bat isolate, have been added to the database (AbuBakar *et al.*, 2004; Chan *et al.*, 2001; Chua *et al.*, 2000, 2002; Harcourt *et al.*, 2005). There are 0–4 aa changes between the V sequences of the different NiV isolates from Malaysia. The isolate from Bangladesh (GenBank accession no. AAY43918; Harcourt *et al.*, 2005) shows considerable variation (up to 53 aa changes, i.e. ∼10 %, throughout the V protein sequence) compared with the Malayan isolates. However, in all of the NiV V sequences, including the bat isolate, and also in the V sequence of the more distantly related HeV, the glycine at position 125 is conserved, in spite of the overall variability of this sequence. Sequence comparison also revealed a single amino acid difference, at position 206 (indicated as a filled box in Fig. 1b[Fig f1]), between the human isolate from Malaysia (the CDC isolate) and the Malaysian bat isolate (GenBank accession no. AAM13407; Chua *et al.*, 2002). Although this mutation (P206L) does not lie within the immediate STAT-binding regions, nor within any other identified motif, the substitution of a proline residue could potentially have a dramatic effect on the functionality of the protein. However, when we introduced the P206L mutation into V*(CDC), the resulting protein V(CDC-P206L) inhibited IFN signalling as efficiently as V*(CDC) in human as well as in bat cells (Fig. 3c[Fig f3]).

The ability to circumvent the IFN response is one of many factors that can influence the host range of a virus. In this study, the V protein of wild-type NiV proved to be functional as an antagonist of IFN signalling in cells from seven different species. Although these experiments only considered the functionality of V *in vitro*, reports on experimental and field infections by other groups confirm that NiV is able to infect and replicate in animals from a broad range of species. Given these observations, the ability of NiV to block IFN signalling does not appear to be a major constraint that prevents this virus from crossing species barriers.

## Figures and Tables

**Fig. 1. f1:**
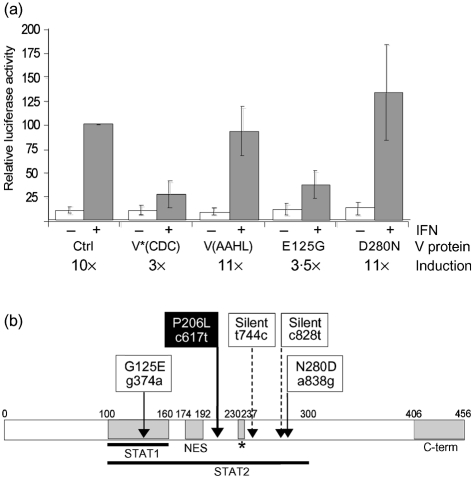
NiV V(AAHL) inhibitory activity is disrupted by a point mutation. (a) Vero cells were transfected with expression vectors for myc-tagged NiV V variants (as indicated) or empty pEF.plink2 expression vector (Ctrl). Cells were also transfected with an IFN-*α*/*β*-responsive luciferase reporter plasmid [p(9–27ISRE)4tkΔ(−39)lucter] and a housekeeping-controlled *β*-galactosidase reporter plasmid (pJATlacZ) (Didcock *et al.*, 1999). Cells were stimulated with 1.8×10^4^ IU IFN-*α* ml^−1^ (Roferon-A; Roche Diagnostics) (+) or left untreated (−), and 4–6 h later were lysed and assayed for luciferase and *β*-galactosidase activity. Luciferase values were normalized to *β*-galactosidase values and the results are shown as means±sd obtained from five independent transfections. Means of induction factors (induced/uninduced activity) are indicated below the graph. (b) Schematic representation of V(CDC) with STAT1- and STAT2-binding regions. Shaded areas indicate (from left to right) the STAT1-binding region, the nuclear export signal (NES), essential residues interacting with STAT2 (*) and the conserved C-terminal domain (C-term). Numbers above the bar represent amino acid positions. Nucleotide and amino acid differences in V(AAHL) are indicated in open boxes. The P206L mutation (see Fig. 3) is indicated in the filled box. Upper case is used for amino acid residues and lower case for nucleotides.

**Fig. 2. f2:**
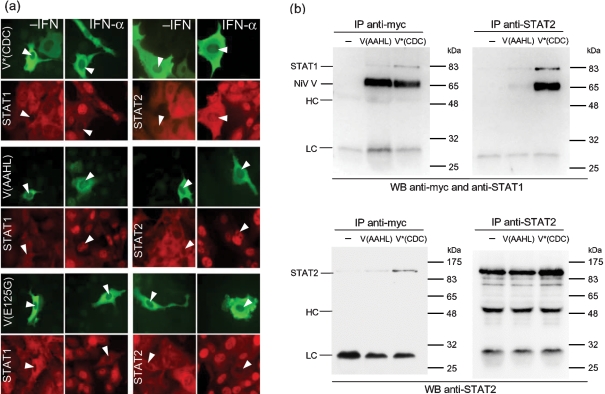
NiV V(AAHL) does not interact with STAT1 or STAT2. (a) Immunofluorescence. 2fTGH cells were transfected with myc-tagged V(AAHL), V*(CDC) or V(AAHL)-E125G expression constructs and stimulated for 70 min with 1.8×10^4^ IU IFN-*α* ml^−1^ (Roferon-A; Roche Diagnostics). Cells were fixed and stained with antibodies against the myc tag (green fluorescence) and against either STAT1 (red fluorescence, left panels) or STAT2 (red fluorescence, right panels) as indicated. (b) Co-immunoprecipitation. 293 cells were transfected with expression constructs encoding STAT1 and STAT2 and either myc-tagged V(AAHL) or V*(CDC). Cells were lysed at 48 h post-transfection and complexes containing the V and STAT proteins were precipitated from the lysates using antibodies against either STAT2 or the myc tag, as indicated above each panel. The precipitates were analysed by Western blotting with antibodies detecting either STAT1 and the myc tag or STAT2, as indicated below the panels. The lower right panel confirms efficient precipitation with the anti-STAT2 antibody in all three lysates. HC, Antibody heavy chain; LC, antibody light chain.

**Fig. 3. f3:**
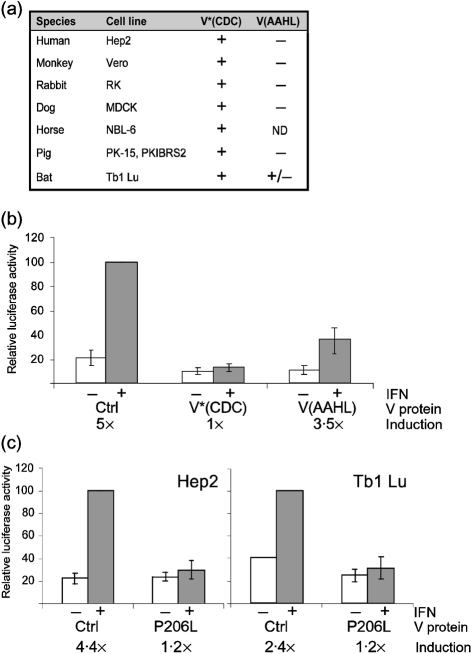
Inhibition of IFN signalling in cells of different species. (a) Summary of signalling results. IFN-*α*/*β* signalling assays were carried out as described. +, Inhibition of signalling; −, failure to inhibit signalling. Results were classified as positive (+) when the induction factor was reduced to 30 % or lower compared with the negative control and the value of the stimulated sample was reduced to 25 % or lower compared with the negative control. (b) Details of the results for Tb1 Lu cells given in (a). Instead of commercial IFN-*α*, these cells were stimulated with purified and UV-inactivated supernatant from Tb1 Lu cells infected with rSV5VΔC (He *et al.*, 2002), a strong inducer of IFN production. Results are shown as means±sd from six independent transfections. Means of induction factors (induced/uninduced activity) are indicated below the graph. (c) Comparison of inhibition of IFN signalling by NiV V(CDC)-P206L in Hep2 and Tb1 Lu cells. Results are shown as means±sd from six (Hep2) or three (Tb1 Lu) independent transfections. Means of induction factors (induced/uninduced activity) are indicated below the graph.
